# Evaluating the Burstlet Theory of Inspiratory Rhythm and Pattern Generation

**DOI:** 10.1523/ENEURO.0314-19.2019

**Published:** 2020-01-13

**Authors:** Prajkta S. Kallurkar, Cameron Grover, Maria Cristina D. Picardo, Christopher A. Del Negro

**Affiliations:** Department of Applied Science, Integrated Science Center, Williamsburg, VA 23185

**Keywords:** breathing, central pattern generator, preBötzinger complex, respiration

## Abstract

The preBötzinger complex (preBötC) generates the rhythm and rudimentary motor pattern for inspiratory breathing movements. Here, we test “burstlet” theory (Kam et al., 2013a), which posits that low amplitude burstlets, subthreshold from the standpoint of inspiratory bursts, reflect the fundamental oscillator of the preBötC. In turn, a discrete suprathreshold process transforms burstlets into full amplitude inspiratory bursts that drive motor output, measurable via hypoglossal nerve (XII) discharge *in vitro*. We recap observations by Kam and Feldman in neonatal mouse slice preparations: field recordings from preBötC demonstrate bursts and concurrent XII motor output intermingled with lower amplitude burstlets that do not produce XII motor output. Manipulations of excitability affect the relative prevalence of bursts and burstlets and modulate their frequency. Whole-cell and photonic recordings of preBötC neurons suggest that burstlets involve inconstant subsets of rhythmogenic interneurons. We conclude that discrete rhythm- and pattern-generating mechanisms coexist in the preBötC and that burstlets reflect its fundamental rhythmogenic nature.

## Significance Statement

Breathing movements depend on a neural rhythm and rudimentary motor pattern. Microcircuits of the brainstem preBötzinger complex (preBötC) produce both, but by unknown mechanisms that prove refractory to canonical explanations. Inspired by an unconventional proposal that rhythm and motor pattern are separable processes (Kam et al., 2013a), we replicated their findings that rhythmicity in local preBötC microcircuits can occur independently without obligatory neural bursts that generate motor output. The rhythm is voltage dependent and the constituent interneurons change from cycle to cycle. These results suggest that breathing rhythm is attributable to recurrent excitation among interneurons that discretely trigger neural bursts and motor output. The preBötC, previously considered uniquely rhythmogenic, contains rhythm- and pattern-generating microcircuits.

## Introduction

Breathing is a vital rhythmic behavior. Inspiration, the inexorable active phase of the breathing cycle, originates from the preBötzinger complex (preBötC) in the lower medulla (Smith et al., 1991; Del Negro et al., 2018). preBötC interneurons generate rhythmic activity and project to cranial and spinal premotor and motor neurons that drive inspiratory muscles. However, the neural mechanisms that give rise to inspiratory rhythm and motor pattern are unclear. These mechanisms can be studied in reduced preparations that isolate the preBötC and cranial hypoglossal (XII) inspiratory motor circuits, and thus provide an experimentally advantageous minimal breathing-related microcircuit *in vitro*. Here, we disentangle neural mechanisms intrinsic to the preBötC that engender both inspiratory rhythm and fundamental aspects of the motor pattern.

We stipulate: preBötC bursts propel inspiratory activity to premotor and motor neurons thus they are important for motor pattern. However, are preBötC bursts rhythmogenic? The following observations cast doubt on that premise. The magnitude of inspiratory bursts (in the preBötC and motor output) can be diminished while only minimally affecting their frequency (Johnson et al., 2001; Del Negro et al., 2002; Peña et al., 2004; Pace et al., 2007). Also, manipulating network excitability affects the frequency, but not the magnitude of preBötC inspiratory bursts and motor output (Del Negro et al., 2009; Sun et al., 2019). It thus appears that two discrete phenomena emanate from the preBötC: a fundamental rhythm (whose frequency is adjustable) and a rudimentary pattern consisting of bursts that drive motor output. These appear to be separable processes as codified by Kam and Feldman in their *burstlet* hypothesis (Kam et al., 2013a; Feldman and Kam, 2015). To describe one cycle, preBötC neurons experience a ramp-like depolarization lasting 100–400 ms, which reflects recurrent excitatory synaptic activity among constituent rhythmogenic neurons (Smith et al., 1990; Rekling et al., 1996; Kam et al., 2013b). This is called the preinspiratory phase because it precedes, and ordinarily leads to, the inspiratory burst. However, Kam and Feldman showed that preinspiratory activity can be divorced from inspiratory bursts by lowering the neural excitability. What often remains is preBötC network activity matching that of the preinspiratory phase but absent the burst; they dubbed these events *burstlets* (Kam et al., 2013a).

Here, we test the burstlet hypothesis of inspiratory rhythm and pattern generation by explicitly or conceptually repeating Kam et al's (2013a) experiments. We, too, detected preBötC burstlets absent XII output, which were distinct from larger amplitude preBötC bursts accompanied by XII output. Manipulations that lower neural excitability increase the prevalence of burstlets relative to bursts. Composite preBötC rhythm depends on cellular excitability because manipulations of external K^+^ concentration control its frequency. Intracellular recordings and photonic imaging of preBötC inspiratory neurons demonstrate that the burstlets occur in subsets of preBötC neurons, not the entire rhythmogenic population that participates in bursts. Our results also support the fundamental tenet of burstlet theory that preinspiratory activity and burstlets reflect a common rhythmogenic mechanism, and that a threshold process causes burstlets (i.e., preinspiratory activity) to trigger bursts and subsequent motor output. These results imply that pattern generation, although a distinct process from rhythm generation, starts from the preBötC core microcircuit.

## Materials and Methods

The Institutional Animal Care and Use Committee at William & Mary approved these protocols, which conform to the policies of the Office of Laboratory Animal Welfare (National Institutes of Health) and the guidelines of the National Research Council (National Research Council, 2011). Mice were housed in colony cages on a 14/10 h light/dark cycle with controlled humidity and temperature at 23°C and were fed *ad libitum* on a standard commercial mouse diet (Teklad Global Diets, Envigo) with free access to water.

### Mice

preBötC field recording experiments employed CD-1 mice (Charles River). Whole-cell recordings employed CD-1 mice as well as mice with Cre-dependent expression of fluorescent Ca^2+^ indicator GCaMP6f dubbed Ai148 by the Allen Institute (RRID:IMSR_JAX:030328; Daigle et al., 2018). We crossed homozygous *Dbx1^Cre^* (Bielle et al., 2005) females with Ai148 males. We refer to their offspring as Dbx1;Ai148 mice. Newborn Dbx1;Ai148 pups express GCaMP6f in neurons derived from progenitors that express the embryonic transcription factor developing brain homeobox 1 (*Dbx1*).

### Slice preparations

Neonatal mice (P0–P4) of both sexes were anesthetized by hypothermia and killed by thoracic transection. Brainstems were removed in cold artificial CSF (ACSF) containing the following: 124 mM NaCl, 3 mM KCl, 1.5 mM CaCl_2_, 1 mM MgSO_4_, 25 mM NaHCO_3_, 0.5 mM NaH_2_PO_4_, and 30 mM dextrose, which we aerated with 95% O_2_ and 5% CO_2_. Brainstems were then glued to an agar block with the rostral side up. We cut a single 450- to 500-µm-thick transverse medullary slice with the preBötC at the rostral surface. The position of the preBötC was benchmarked according to neonatal mouse preBötC atlases (Ruangkittisakul et al., 2011, 2014).

### Electrophysiology

Slices were held in place and perfused with ACSF (∼28° C) at 2–4 ml min^−1^ in a recording chamber on a fixed-stage upright microscope. The external K^+^ concentration, i.e., [K^+^]_o_, in the ACSF was initially raised to 9 mM, which facilitates robust rhythm and motor output in slices (Funk and Greer, 2013).

Population activity from preBötC interneurons and XII motor neurons was recorded using suction electrodes fabricated from borosilicate glass pipettes (OD: 1.2 mm, ID: 0.68 mm). preBötC field recordings were obtained by placing the suction electrode over the rostral face of the preBötC at the surface of the slice. XII motor output was recorded from XII nerve rootlets, which are retained in slices. Signals were amplified by 20,000, band pass filtered at 0.3–1 kHz, and then RMS smoothed using a differential amplifier (Dagan Instruments). Smoothed signals were used for display and quantitative analyses.

We used an EPC-10 patch-clamp amplifier (HEKA Instruments) for whole-cell current-clamp recordings. Patch pipettes were fabricated from borosilicate glass (OD: 1.5 mm, ID: 0.86 mm) to have tip resistance of 4–6 MΩ. The patch pipette solution contained the following: 140 mM K-gluconate, 5 mM NaCl, 0.1 mM EGTA, 10 mM HEPES, 2 mM Mg-ATP, and 0.3 mM Na_3_-GTP. We added 50 µM Alexa Fluor 488 hydrazide dye (A10436, Life Technologies) for visualization after whole-cell dialysis. Whole-cell recordings were made from preBötC inspiratory neurons selected visually based on rhythmic fluorescence changes in Dbx1;Ai148 mice. In CD-1 mice, we only collected whole-cell data from preBötC inspiratory neurons that were active in sync with XII motor output. Membrane potential trajectories were low-pass filtered at 1 kHz and digitally recorded at 4 kHz using a PowerLab data acquisition system, which includes a 16-bit analog-to-digital converter and LabChart v7 software (ADInstruments).

We modified [K^+^]_o_ in the ACSF from 9 to 3 mM to modulate the network excitability. Each trial consisted of a sequence of non-contiguous [K^+^]_o_ levels selected randomly in descending order.

Low-amplitude activity in preBötC field recordings was classified as a burstlet if it met these two criteria: the peak of preBötC activity exceeded the mean of the distribution of baseline noise by 2*SD, and there was negligible concurrent activity in the XII root recording. The mean and SD of baseline noise were obtained by sampling every data point during a sliding 120-s window, constructing a histogram of baseline noise, and fitting that distribution with a Gaussian function to obtain the mean and SD.

A sigh burst in the preBötC field recording was distinguished from an inspiratory burst if it met these three criteria: the area of the putative sigh burst exceeded the mean area of all inspiratory bursts by one SD; the cycle period of the putative sigh bursts measured 1–4 min and not outside this range; and the putative sigh burst was followed by a prolonged inter-event interval >1.3 times the average inspiratory cycle time for six consecutive cycles preceding a putative sigh burst (Lieske et al., 2000; Ruangkittisakul et al., 2008; Borrus et al., 2019).

For field recordings, we measured the amplitude (amp) of the preBötC population activity and XII motor output, as well as inspiratory frequency (f; or cycle time). We also measured the rise time, decay time, and duration of burstlets in field recordings. For whole-cell recordings, inspiratory bursts refer to depolarizations with concomitant spiking in preBötC neurons that occur in sync with XII motor output. We measured the amplitude of inspiratory bursts after smoothing to eliminate spikes but preserve the envelope of depolarization. To identify burstlet-like activity in whole-cell recordings, we performed simultaneous field recordings from the contralateral preBötC as well as XII motor output.

We wrote algorithms in MATLAB (RRID:SCR_001622) to calculate the mean frequency as well as the amplitude of bursts and burstlets. The coefficient of variation (CV) of preBötC or XII motor output frequency was calculated as the ratio of SD to the mean frequency.

For all intracellular and two-photon recordings, a neuron that participates in an inspiratory burst is referred as a burst-active neuron and a neuron that participates in a burstlet, is referred as a burstlet-active neuron.

Cycle-triggered averages were calculated and plotted in IgorPro (v.8, RRID:SCR_000325) using the onset of XII output as the event trigger for averaging preBötC inspiratory bursts; the onset of the burstlet itself served as the event trigger for averaging burstlets. We obtained the depolarization rate (V/s) of the event-triggered averages as the quotient of event amplitude and the elapsed time for that event to reach its peak amplitude. For preinspiratory activity, event amplitude was calculated as the absolute difference between the baseline level of activity and the level of activity in the field recording at the onset of XII motor output. For bursts, event amplitude was calculated as the difference between peak amplitude and field amplitude at the onset of XII motor output (that procedure omits the amplitude portion attributable to the preinspiratory phase). For burstlets, event amplitude was calculated as the difference between peak amplitude and baseline at the onset of the burstlet.

### Two-photon imaging

We imaged intracellular Ca^2+^ in neurons contained in slices from Dbx1;Ai148 mice using a multi-photon laser-scanning microscope (Thorlabs) equipped with a water immersion 20x, 1.0 numerical aperture objective. Illumination was provided by an ultrafast tunable laser with a power output of 1050 mW at 970 nm, 80-MHz pulse frequency, and 100-fs pulse duration (Coherent Chameleon Discovery). We scanned Dbx1;Ai148 mouse slices over the preBötC and collected time series images at 32 Hz. Each frame reflects one-way raster scans with a resolution of 256 × 256 pixels (116 × 116 µm). Fluorescence data were collected using Thorlabs LS software and then analyzed using Fiji (Schindelin et al., 2012; Schneider et al., 2012), MATLAB, and IgorPro.

Regions of interest (ROIs) representing individual *Dbx1*-derived preBötC neurons were detected using MATLAB. First we found the set of collective inspiratory bursts in the time series from each Dbx1;Ai148 slice by averaging the fluorescence intensity of all pixels for each frame; fluorescence peaks are easily detectable periodic events. The collection of cycle periods is normally distributed; the 95% confidence intervals (CIs) are defined by the mean cycle period ± 2*SD.

Next, we down-sampled the planar resolution of our stack of images by 2^n^ (*n* ≥ 1). The mean fluorescence of the constituent pixels was assigned to each composite pixel. We performed temporal fast Fourier transforms (FFTs) on the composite pixels. The maximum FFT value within the 95% CIs for slice frequency (determined above) was then mapped to a corresponding position in a new processed two-dimensional image. This method quantifies how strongly a composite pixel changes fluorescence at frequencies that correspond to inspiratory rhythm. After having created the complete processed image, we computed the mean and SD for FFT values associated with all composite pixels. Any composite pixel with intensity less than mean + 2*SD was set to zero. Any contiguous remaining pixel sets (whose diameter exceeds 6 µm) were retained as ROIs.

We then applied the set of ROIs to analyze Ca^2+^ transients in the original fluorescence imaging stack using the equation (F_i_ – F_0_)/F_0_, i.e., ΔF/F_0_, where F_i_ is the instantaneous average fluorescence intensity of all the pixels in a given ROI and F_0_ is the average fluorescence intensity of all the pixels within the same ROI averaged over the entire time series. Finally, we smoothed the ΔF/F_0_ time series with a forward moving average with a window of four time points.

### Statistics

All the null hypothesis statistical tests were calculated using Prism (v.8, RRID:SCR_002798). Changes in the frequency and amplitude of preBötC field activity and XII output as a function of [K^+^]_o_ were evaluated using linear regression. Changes in the rise time, decay time, and duration of burstlets were evaluated using linear regression. We compared group means using either Student’s paired *t* test or repeated measures one-way ANOVA, applying Holm–Sidak’s multiple comparison test *post hoc*. We compared the variability of frequency of preBötC events and XII motor output using a Kruskal–Wallis test, applying Dunn’s multiple comparison test *post hoc*. We compared the frequency of preBötC events using Welch’s *t* test.

## Results

### preBötC generates bursts at high levels of excitability but burstlets appear as excitability decreases

**Figure 1.**
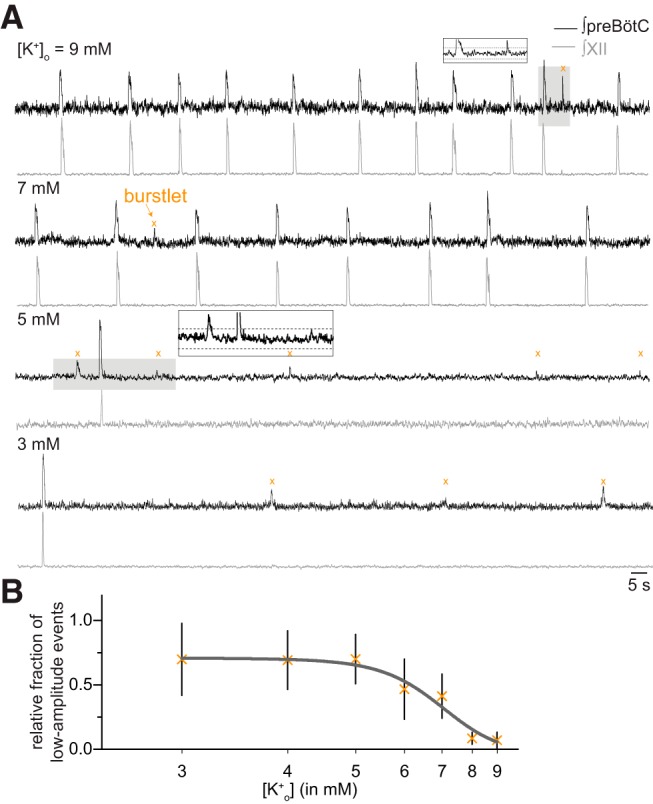
preBötC field and XII recordings demonstrate inspiratory burst and “burstlet” rhythms. ***A***, preBötC field (black) and XII (gray) recordings at different levels of [K^+^]_o_. Insets show expanded traces from gray boxes. Dashed lines in the insets mark the 95% CIs. Orange × symbols indicate burstlets. Time calibration applies to all traces. ***B***, The relative fraction of burstlets in preBötC field recordings as a function of [K^+^]_o_. Vertical bars show SD.

We manipulated excitability by varying [K^+^]_o_ by integer units between 9 and 3 mM. At high [K^+^]_o_ (e.g., 9 or 7 mM in Fig. 1*A*), we observed mostly burst events, defined by peaks of activity in the preBötC field recording with coincident XII output. Nevertheless, we also observed events in the preBötC field recording whose amplitudes measured 15–65% of the bursts and occurred without coincident XII discharge (Fig. 1*A*).

We detected and then studied low-amplitude preBötC events whose peaks exceeded the 95% CIs of baseline noise (Fig. 1*A*, insets). That detection process ensures that low-amplitude events are unlikely (with probability <0.05) to be ordinary uncoordinated fluctuations of neural activity. The alternative is that these low-amplitude preBötC events reflect coherent network activity.

If the low-amplitude events reflect burstlets as defined by Kam and Feldman (Kam et al., 2013a), then they should be more abundant at low levels of excitability where the collective activity of rhythmogenic neurons may not reach the threshold for burst generation. Visual inspection of the traces in Figure 1*A* shows that to be the case, i.e., low-amplitude events devoid of XII output are more abundant at 5 and 3 mM [K^+^]_o_ compared to 9 and 7 mM [K^+^]_o_.

At 3 mM [K^+^]_o_, 70 ± 3% (*n* = 12 slices) of detected preBötC events occurred without concomitant XII output. We quantified the relative abundance of low-amplitude versus burst events for the entire data set (Fig. 1*B*, *n* = 19 slices). At incrementally higher [K^+^]_o_ levels, the relative fraction of low-amplitude events decreases in a sigmoidal fashion such that they comprise only 5.2 ± 6.3% (*n* = 19 slices) of the preBötC events at 9 mM. We conclude that low-amplitude preBötC events, absent motor output, reflect burstlets as defined previously (Kam et al., 2013a).

### Burst-burstlet and sigh rhythmic frequencies vary as a function of network excitability

**Figure 2.**
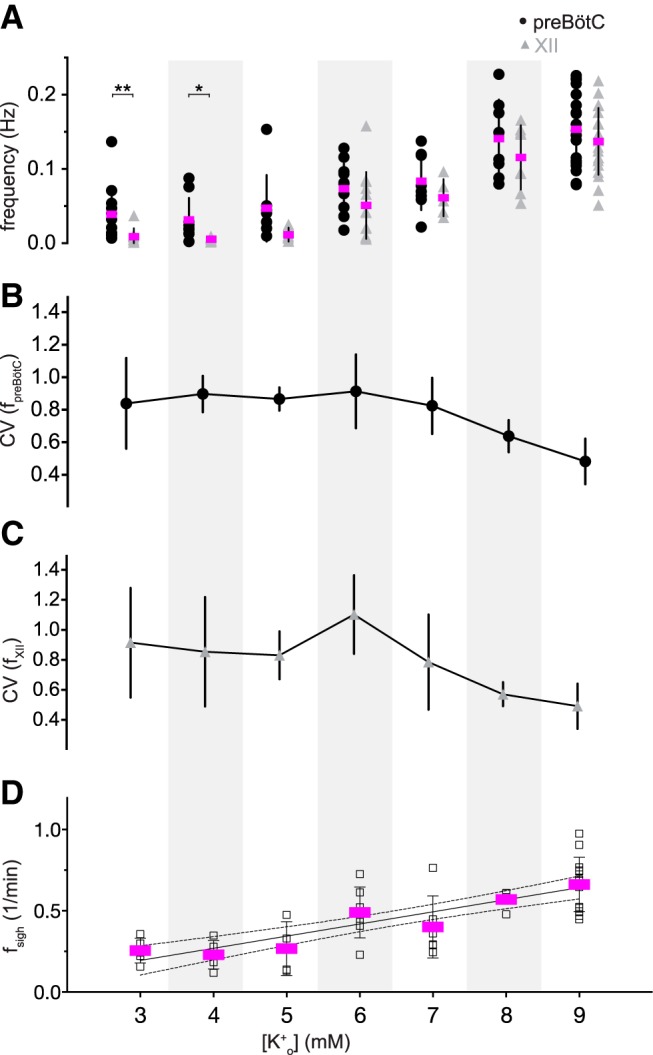
Frequency and variability of preBötC composite rhythm, XII motor output, and sigh rhythm. ***A***, Frequency of composite rhythm (f_preBötC_, filled circles) and XII motor output (f_XII_, gray triangles) as a function of external K^+^ concentration, i.e., [K^+^]_o_. The frequency from each slice preparation is shown with mean frequency (magenta) and SD (vertical lines). Statistical significance is indicated by symbols as *p < 0.05, **p < 0.01. ***B***, ***C***, Mean CV for composite (filled circles) and XII (gray triangles) rhythms as a function of [K^+^]_o_. Vertical bars show SD. ***D***, Frequency of sigh rhythm (f_sigh_, black unfilled squares). The frequency from each slice preparation is shown with mean frequency (magenta) and SD (vertical lines). Light gray background shading was applied to differentiate [K^+^]_o_ levels.

We measured the frequency of preBötC rhythm (f_preBötC_, which we refer to as composite rhythm because the measured events consist of either bursts or burstlets) and XII motor output (f_XII_) at different [K^+^]_o_ levels (Fig. 2*A*) similar to Kam et al. (2013a). They measured the f_preBötC_ and f_XII_ at three discrete [K^+^]_o_ levels (3, 6, and 9 mM) and reported significantly lower f_XII_ at 3 mM compared to either 6 or 9 mM. Regarding f_preBötC_, it was lower at 3 mM, yet there was no difference between the f_preBötC_ measured at 6 vs 9 mM (Kam et al., 2013a, see their Fig. 1*A* and Table 1). Those data do not resolve the relationship between composite rhythm and network excitability so we measured rhythmic activity at all integer [K^+^]_o_ levels between 3 and 9 mM. f_preBötC_ and f_XII_ increased linearly as the excitability increased (in this report: Fig. 2*A*, Table 1, which reports linear regression tests). Additionally, f_preBötC_ and f_XII_ differed significantly at both 3 and 4 mM [K^+^]_o_ (Welch’s *t* test, *p* = 0.009 and *p* = 0.031, respectively) which maps to the portion of the curve in Figure 1*B*, where the relative fraction of burstlets plateaus at 70 ± 30% (*n* = 12 slices) and thus explains the relative sparsity of XII events compared to preBötC burstlets. Manipulations of network excitability influence the frequency of composite rhythm and the relative fraction of burstlets that comprise it.

**Table 1.** Linear regression analyses of the effects of [K^+^]_o_ on the frequency and amplitude of preBötC events and XII motor output

	Slope (Hz/mM or 1/min mM or V/mM)	*p* value	*R* ^2^	Figure
preBötC composite frequency (Hz)	0.021	3.0E-14	0.55	2*A*
XII motor output frequency (Hz)	0.024	2.0E-17	0.68	2*A*
Sigh frequency (1/min)	0.074	1.9E-8	0.53	2*D*
Burst amplitude (V)	0.049	0.673	0.002	3*A*
Burstlet amplitude (V)	0.179	0.0001	0.19	3*B*
XII amplitude (V)	0.148	0.497	0.01	3*C*

[K^+^]_o_ was changed from 3 to 9 mM in 1 mM steps. All results were analyzed using linear regression analysis. Number of slices (*n*) at each [K^+^]_o_ (mM, *n*): (3, 12); (4, 9); (5, 8); (6, 12); (7, 8); (8, 8); (9, 19).

Changes in the network excitability affect the periodic variability of XII motor output (Del Negro et al., 2009). We reexamined that principle and further tested the variability of the composite preBötC rhythm (Fig. 2*B*). The variability of f_XII_, quantified by CV, peaked at ∼1.1 when [K^+^]_o_ was 6 mM (Fig. 2*C*; Table 3). In contrast, the CV of f_preBötC_ remained between 0.9 and 0.7 over low to medium levels of excitability (3–6 mM [K^+^]_o_) without peaking at 6 mM [K^+^]_o_ (Fig. 2*B*; Table 3). These data suggest that peak CV of f_XII_ at 6 mM [K^+^]_o_, an intermediate level of excitability, is not attributable to instability in the preBötC rhythm. It rather reflects the equal probability of evoking either burstlets (absent XII output) and bursts with XII output (Fig. 1*B* or Kam et al., 2013a, their Fig. 2), which makes the periodic XII output more variable than preBötC activity.

The variability of f_preBötC_ and f_XII_ are lowest at 9 mM [K^+^]_o_ (Fig. 2*B*,*C*; Table 3). ACSF containing 9 mM [K^+^]_o_ represents the empirically determined ideal conditions for rhythmically active slices (Funk and Greer, 2013; Smith et al., 1991) where the likelihood of burstlets is minimal (Fig. 1*B*; Kam et al., 2013a, their Table 1).

We also examined the frequency of the sigh rhythm (f_sigh_), which increased linearly as the network excitability increased by means of [K^+^]_o_ (Fig. 2*D*; Table 1).

**Figure 3.**
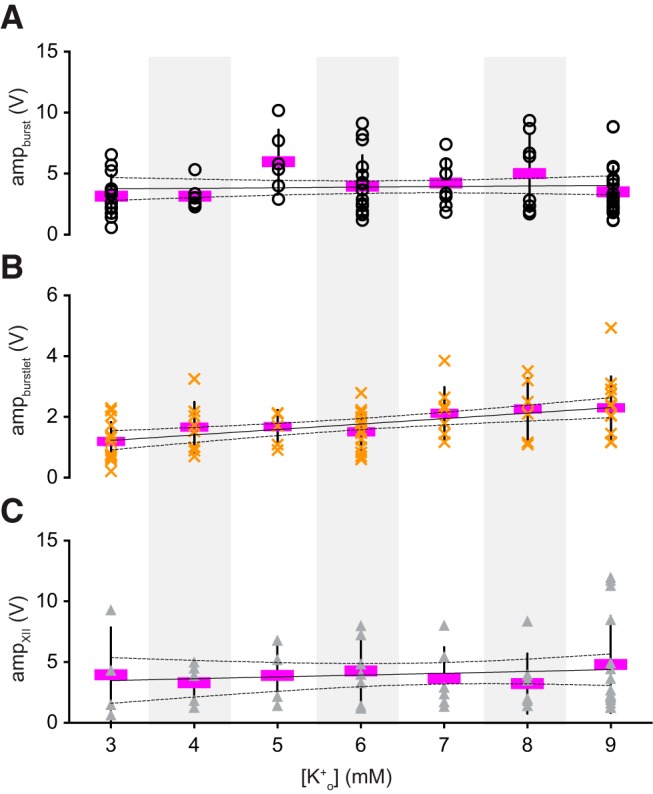
Amplitude of preBötC events and motor output. ***A–C***, Amplitude of inspiratory bursts (amp_burst_, open circles), burstlets (amp_burstlet_, orange × symbols), and XII output (amp_XII_, gray triangles) as a function of external K^+^ concentration, i.e., [K^+^]_o_. The amplitude of each slice preparation is shown with mean amplitude (magenta) and SD (vertical lines). Linear regression lines (solid) and 95% CIs (dashed) are shown. Light gray background shading was applied to differentiate [K^+^]_o_ levels.

We also examined the amplitude of bursts, burstlets, and XII output. The amplitude of preBötC bursts and XII output were invariable over all [K^+^]_o_ levels (Fig. 3*A*,*C*), confirmed using linear regression tests (Table 1). However, the burstlet amplitude increased from 1.2 ± 0.6 mV at 3 mM [K^+^]_o_ (*n* = 12 slices) to 2.3 ± 1.0 mV (*n* = 13 slices) at 9 mM K^+^ (Fig. 3*B*), which linear regression showed was unlikely to occur by chance if the slope of burstlet amplitude versus [K^+^]_o_ was actually zero (*p* = 0.001; Table 1). These results show that burstlet amplitude depends on network excitability whereas preBötC burst and XII motor output amplitudes do not.

We measured the rise time, decay time, and duration of burstlets, but there were no trends across [K^+^]_o_ levels, confirmed using linear regression (Table 2).

**Table 2.** Effects of [K^+^]_o_ on the rise time, decay time, and duration of burstlet

[K^+^]_o_ (mM)	Rise time (ms)	Decay time (ms)	Duration (ms)
3	124.9 ± 70.5	317.9 ± 184.8	400.1 ± 165.4
4	173.1 ± 97.4	262.1 ± 164.1	394.1 ± 220.8
5	130.7 ± 79.5	236.6 ± 83.5	375.2 ± 163.2
6	208.4 ± 105.3	232.8 ± 92.9	352.7 ± 129.2
7	173.9 ± 39.9	246 ± 89.9	350.2 ± 94.8
8	135.3 ± 59.7	255.2 ± 125.8	361.4 ± 132.3
9	155.6 ± 77.8	198.2 ± 66.4	310.3 ± 86
Linear regression test			
Slope (ms/mM)	1.25	–13.75	–13.60
*p* value	0.067	3.199	2.245
*R* ^2^	0.001	0.060	0.043

The rise time, decay time, and duration of burstlet are reported as mean ± SD. All the results were analyzed using linear regression analysis. Number of slices (*n*) at each [K^+^]_o_ (mM, *n*): (3, 8); (4, 9); (5, 6); (6, 8); (7, 8); (8, 8); (9, 14).

**Table 3.** Effects of [K^+^]_o_ on the variability of frequency of preBötC composite rhythm and XII motor output

	H (df)	*p* value	*Post hoc* analysis	Figure
CV (f_preBötC_)	H(7) = 19.61	0.003	6 vs 9 mM [K^+^]_o_: 0.005	2*A*
CV (f_XII_)	H(7) = 29.01	1.408E-5	6 vs 8 mM [K^+^]_o_: 0.039 6 vs 9 mM [K^+^]_o_: 7.968E-7	2*B*

The reported Kruskal–Wallis test statistic (H), degrees of freedom (df), and *p* values compare frequency variability of composite rhythm and XII motor output as [K^+^]_o_ is changed from 3 to 9 mM. *Post hoc* analysis (calculated using Dunn’s test) report *p* values for the group that were statistically significant.

### Burstlets evolve bilaterally and underlie the pre-inspiratory phase of preBötC bursts

**Figure 4.**
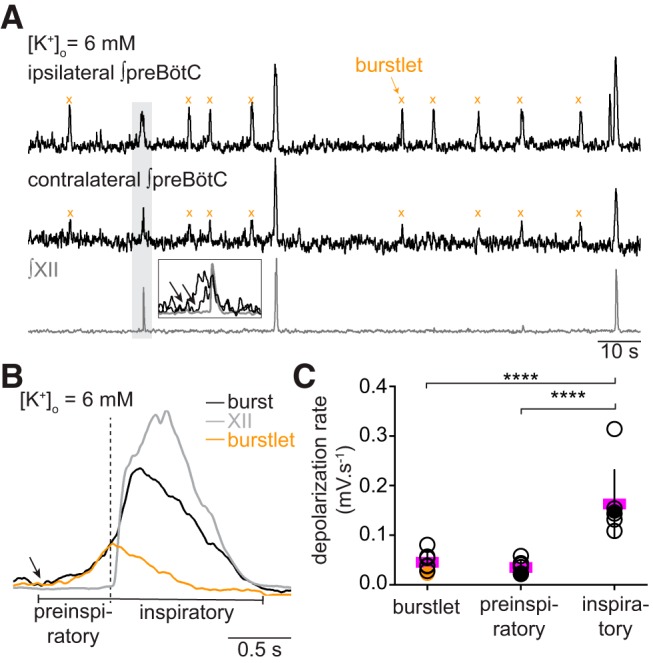
Burstlets are bilaterally synchronous and makeup the preinspiratory phase of bursts. ***A***, Bilateral field recordings of the preBötC, with XII output (gray). ***B***, Cycle-triggered averages of burstlets (orange), inspiratory bursts (black), and XII output (gray). The onset of preinspiratory preBötC activity and burstlets are marked by an arrow; their peaks occur at the onset of XII motor output, marked by vertical dashed line. For inspiratory bursts, the onset of XII motor output marks the onset of the event. ***C***, Plot of rising slope of burstlets, the preinspiratory phase of bursts, and the inspiratory burst itself. The depolarization rate for each slice is shown with individual symbols (filled symbols are shown in the example in ***B***); the mean shown in magenta. Asterisks indicate statistical significance at *p* < 0.0001.

If burstlets reflect coherent preBötC rhythmicity, then they should be bilaterally synchronous. To test that prediction, we recorded preBötC activity from both sides of slices along with XII motor output (Fig. 4*A*); 97 ± 4% and 92 ± 7% of preBötC burstlets were bilaterally synchronous at 6 and 3 mM [K^+^]_o_, respectively (*n* = 4 slices). The bilateral preBötC bursts commence ∼400 ms before the onset of XII motor output (Fig. 4*A*, inset), which is considered the preinspiratory phase and the hallmark of rhythm generation (Smith et al., 1990; Rekling et al., 1996; Kam et al., 2013a; Feldman and Kam, 2015; Del Negro et al., 2018).

If burstlets reflect the preinspiratory component of inspiratory bursts, as proposed by Kam and Feldman, then their trajectories should look alike when superimposed. At 6 mM [K^+^]_o_, the rising phase of the burstlet resembles the preinspiratory phase of the inspiratory burst (Fig. 4*B*). We compared the depolarization rates of the rising phase of burstlets, the preinspiratory phase of inspiratory bursts, and the rising phase of inspiratory bursts. The rising phase of burstlets is comparable to the rising phase of the preinspiratory activity, but the rising phase of both burstlets and preinspiratory activity are incommensurate with the rising phase of inspiratory bursts (one-way ANOVA, *F*_(2,12)_ = 33.76, *p* = 1.2E-5; burstlet vs preinspiratory, *p* = 0.577; burstlet vs inspiratory, *p* = 2.5E-5; and preinspiratory vs inspiratory, *p* = 3.7E-5; *n* = 7 slices; Fig. 4*C*). These data suggest that burstlets and the preinspiratory phase of bursts reflect the same underlying process, which is distinct from the process underlying full inspiratory bursts.

### Burstlets are the summation of EPSPs in preBötC neurons

We examined how individual preBötC inspiratory neurons contribute to collective events detected in field recordings (bursts and burstlets) via whole-cell recordings in CD-1 (*n* = 7) and Dbx1;Ai148 mouse (*n* = 3) slices. Dbx1;Ai148 mouse pups express genetically encoded Ca^2+^ reporter GCaMP6f in *Dbx1*-derived preBötC neurons obligatory for breathing rhythmogenesis (Bouvier et al., 2010; Gray et al., 2010; Wang et al., 2014; Cui et al., 2016; Vann et al., 2016, 2018; Baertsch et al., 2018).

**Figure 5.**
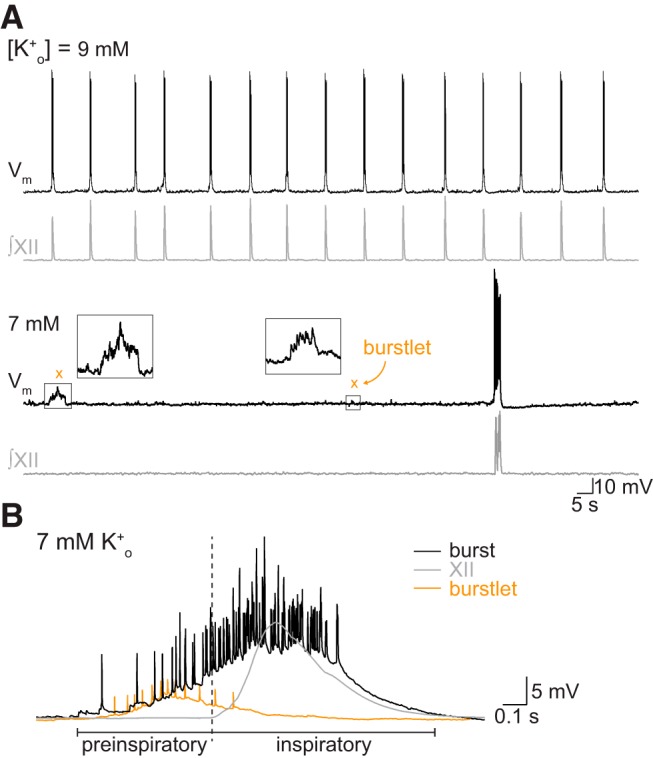
Properties of burstlets recorded intracellularly. ***A***, Whole-cell recording (V_m_, top traces) from an inspiratory preBötC neuron with XII output (gray, lower traces) at 9 and 7 mM external K^+^ concentration, i.e., [K^+^]_o_. Insets magnify burstlets at 7 mM [K^+^]_o_. ***B***, Cycle-triggered averages of burstlets (orange) and inspiratory bursts (black) recorded intracellularly with XII output (gray). Preinspiratory and inspiratory burst phases are marked. ***A***, ***B*** have separate voltage and time calibrations.

In control conditions (9 mM [K^+^]_o_), inspiratory drive potentials synchronized with XII motor output during almost all cycles (96 ± 7%, *n* = 16 preBötC neurons recorded in slices from 10 different animals). We considered those events bursts. We then modified the excitability by changing [K^+^]_o_ to either 6 mM (*n* = 11 neurons in eight slices) or 7 mM (*n* = 6 neurons in three slices). We recorded drive potentials of 6- to 10-mV amplitude that were not accompanied by XII motor output. We considered those events burstlets (Fig. 5*A*).

During bursts, preBötC neurons, from a baseline membrane potential of –60 mV (maintained below the activation threshold of persistent Na^+^ current via bias current), exhibit inspiratory drive potentials exceeding 20 mV and intraburst spiking of 2–17 spikes/burst (∼6–60 Hz). During burstlets, these same neurons exhibit EPSPs that summate during the burstlets (Fig. 5*A*, insets) as well as spikes (Fig. 5*B*, cycle-triggered average from a whole-cell recording). We never observed a preBötC neuron (*n* = 0/17 neurons in 10 slices) that was active during burstlets but not bursts.

Burstlets resemble the preinspiratory phase of bursts. This applies to field recordings (Fig. 4*B*) and whole-cell recordings at both [K^+^]_o_ levels: 7 mM (*n* = 4 out of six neurons) and 6 mM (*n* = 6 out of 11 neurons; Fig. 5*B*). These are the first intracellular recordings to show that burstlets reflect the temporal summation of EPSPs, often crossing threshold to generate repetitive spiking, in preBötC neurons.

### preBötC inspiratory neurons do not participate in every burstlet

**Figure 6.**
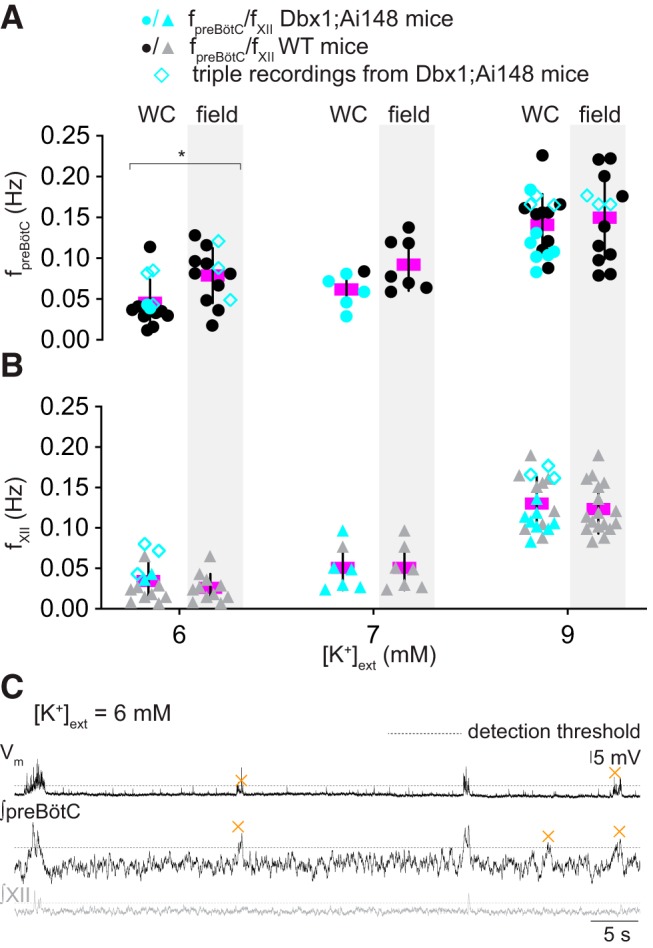
preBötC inspiratory neurons do not participate in every burstlet. ***A***, ***B***, f_preBötC_ (filled circles) and f_XII_ (filled triangles) measured during separate or simultaneous whole-cell and field recordings at three levels of external K^+^ concentration, i.e., [K^+^]_o_: 6, 7, and 9 mM. At each [K^+^]_o_ level, whole-cell (WC) is at left and the preBötC field recording is at right (with light gray background shading). Cyan symbols represent measurements from *Dbx1*-derived preBötC neurons in Dbx1;Ai148 reporter mice. Cyan unfilled diamonds represent measurements from simultaneous triple recording of *Dbx1*-derived preBötC rhythmogenic neuron, preBötC population activity, and XII motor output. The frequency from each slice preparation is shown with mean frequency (magenta) and SD (vertical lines). Asterisk indicates statistical significance at *p* < 0.05. ***C***, Whole-cell recording (V_m_, top trace) from a *Dbx1*-derived inspiratory preBötC neuron with preBötC field recording (∫preBötC, middle trace) and XII (∫XII, bottom trace) at 6 mM K^+^. Dashed lines mark the 95% CI. Orange × symbols indicate burstlets. Vertical calibration only applies to the top trace (V_m_). Time calibration applies to all traces.

Kam et al. (2013a) showed that 89% of the inspiratory preBötC neurons take part in burstlets. We retested that notion by comparing f_preBötC_ and f_XII_ monitored during separate or simultaneous whole-cell and field recordings (Fig. 6*A*,*B*). We predicted that if 89% of inspiratory neurons participate in burstlets, then the frequency of composite rhythm obtained in whole-cell recordings should be comparable to that obtained in field recordings. We found no difference in f_preBötC_ measured via whole-cell and field recordings at 7 or 9 mM [K^+^]_o_. However, at 6 mM [K^+^]_o_, f_preBötC_ was significantly lower in whole-cell recordings compared to field recordings (Fig. 6*A*,*B*; Table 4). Additionally, at all [K^+^]_o_ levels, the f_XII_ calculated in whole-cell recordings and field recordings remain the same. These data imply that relatively fewer inspiratory preBötC neurons are burstlet-active at 6 mM [K^+^]_o_ compared to 7 or 9 mM. Simultaneous triple recordings of *Dbx1*-derived preBötC rhythmogenic neurons, preBötC population activity, and XII motor output demonstrate that individual neurons do not participate in every population burstlet (Fig. 6*C*).

**Table 4.** Comparison of frequencies of preBötC composite rhythm measured in whole-cell recordings and field recordings at 6, 7, and 9 mM [K^+^]_o_

[K^+^]_o_ (mM)	Whole cell (Hz)	Field (Hz)	*p* value
6	0.04 ± 0.03	0.08 ± 0.03	0.04
7	0.06 ± 0.02	0.09 ± 0.03	0.21
9	0.14 ± 0.04	0.14 ± 0.06	0.65

Frequencies are reported as mean ± SD. The reported *p* values (calculated using Welch’s *t* test) compare samples of composite frequency measured from the preBötC in either whole-cell or field recordings.

### Burstlets occur in subsets of inspiratory preBötC neurons

**Figure 7.**
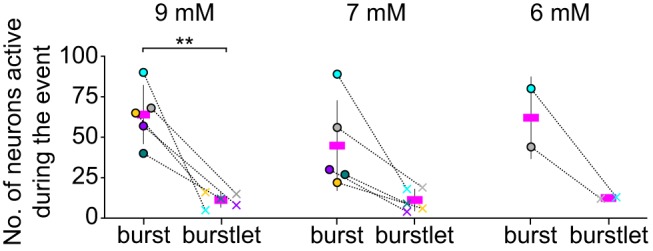
Burstlets occur in subset of preBötC inspiratory neurons. Group data showing the number of neurons active during bursts and burstlets from Dbx1;Ai148 slices at 9, 7, and 6 mM external K^+^ concentration, i.e., [K^+^]_o_. Active neuron counts are illustrated for each slice preparation for bursts (filled circles) and burstlets (× symbols). The dataset is color coded for each slice. Only two slices were sufficiently rhythmically active at 6 mM [K^+^]_o_ to obtain reliable measurements of burstlets within the 2-min recording duration of imaging time series. Asterisks signify statistical significance at *p* < 0.01.

**Figure 8.**
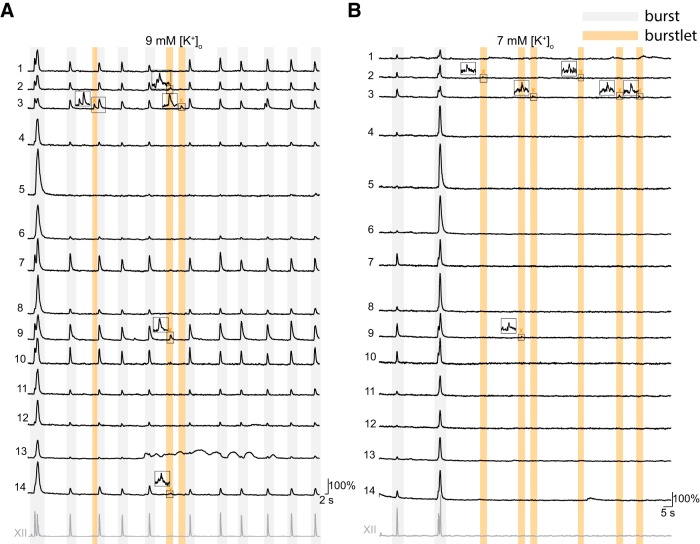
Burstlets occur in dynamic subsets of preBötC inspiratory neurons. Bursts and burstlets in a group of 14 *Dbx1*-derived preBötC neurons in a typical Dbx1;Ai148 mouse slice preparation with inspiratory XII output (lowest traces, gray). Inspiratory cycles are background shaded in light gray. Burstlet cycles are background shaded in light orange. ***A***, ***B*** show population activity in 9 and 7 mM external K^+^ concentration, i.e., [K^+^]_o_, respectively. Insets in ***A***, ***B*** show select burstlets.

To investigate how many and which neurons participate in burstlets, we recorded inspiratory *Dbx1*-derived preBötC neurons in Dbx1;Ai148 slices while simultaneously monitoring XII motor output (Figs. 7, 8). We recorded three to nine imaging planes per preBötC with 12 ± 7 active neurons per plane (range 3–27) for an average of 62 ± 20 inspiratory neurons recorded per Dbx1;Ai148 slice. Then, we manipulated [K^+^]_o_ to examine burst (9 mM; *n* = 6 slices) and burstlet (7 mM, *n* = 6 slices; 6 mM, *n* = 2 slices) rhythms.

At 9 mM [K^+^]_o_, 20 ± 9% of the *Dbx1*-derived inspiratory neurons were active during burstlets (Fig. 7). Additionally, a neuron that was active during one burstlet was not always active during other burstlets (Fig. 8*A*, neurons 2, 3, 9, 14). On changing to 7 mM [K^+^]_o_, 17 ± 15% of the *Dbx1*-derived inspiratory neurons were active during burstlets (Fig. 7). Again, a neuron that participated in one burstlet did not always participate in the next burstlet (Fig. 8*B*, neurons 2, 3, 9). On changing to 6 mM [K^+^]_o_, 23% of *Dbx1*-derived neurons were active during burstlets (Fig. 7). These data show that the subset of *Dbx1*-derived preBötC neurons that participates in burstlets constitutes 17–23% of the population active during inspiratory bursts and that the composition of the burstlet-active subset varies from cycle to cycle.

## Discussion

Inspiratory breathing movements emanate from neural activity in the preBötC but its rhythmogenic mechanisms remain incompletely understood and, some might argue, misunderstood. There appears to be a dichotomy between the mechanisms underlying rhythmogenesis and those governing motor pattern. Here, we investigate this rhythm-pattern dichotomy to unravel the neural mechanisms of preBötC functionality.

### Defunct theories of inspiratory rhythmogenesis and why burstlets are viable explanation

Theories of rhythmogenesis fall into three camps. The first posits a ring of mutually inhibitory neurons that generates sequential phases of the breathing cycle including preBötC inspiratory bursts (Richter, 1982; Smith et al., 2007, 2013; Ausborn et al., 2018). The second theoretical framework emphasizes bursting-pacemaker neurons; the synchronization of pacemakers serves as a template for network activity (Feldman and Cleland, 1982; Johnson et al., 1994; Rekling and Feldman, 1998; Butera et al., 1999a,b; Ramirez et al., 2004). The third theory, dubbed a group pacemaker, posits that recurrent synaptic activity triggers mixed-cationic conductances to produce inspiratory bursts (Rekling et al., 1996; Rekling and Feldman, 1998; Del Negro and Hayes, 2008; Rubin et al., 2009).

Disinhibition of the preBötC (Shao and Feldman, 1997; Brockhaus and Ballanyi, 1998; Janczewski et al., 2013; Sherman et al., 2015; Marchenko et al., 2016; Cregg et al., 2017; Baertsch et al., 2018), as well as attenuation of pacemaker conductances (Del Negro et al., 2002, 2005; Peña et al., 2004; Pace et al., 2007; Koizumi and Smith, 2008) and mixed-cationic conductances (Koizumi et al., 2018; Picardo et al., 2019) neither perturbs the frequency in the predicted manner nor stops breathing *in vivo* or inspiratory rhythms *in vitro*, which falsifies all three rhythmogenic mechanisms. Nevertheless, the key to understanding rhythmogenesis may be found in what these theories get wrong: inextricable neural bursts that culminate the inspiratory phase of the cycle.

To consider the iconoclastic notion of rhythmogenesis in the absence of bursts we, like Kam and Feldman (Kam et al., 2013a; Feldman and Kam, 2015), focus on the preinspiratory phase that ordinarily leads to bursts and motor output. The preinspiratory phase is a hallmark of rhythmogenesis, marking early-activating rhythmogenic interneurons (Onimaru et al., 1987, 1988; Smith et al., 1990; Rekling et al., 1996; Carroll and Ramirez, 2013; Carroll et al., 2013). Concurrent excitation of four to nine preBötC interneurons *in vitro*, by photolytic glutamate uncaging (Smith et al., 1990; Rekling et al., 1996; Kam et al., 2013b; Sun et al., 2019), can effectively trigger a preBötC network burst after a latency of 100–400 ms, similar to the duration of the preinspiratory phase. There are two important take-aways: first, small numbers (<10) of coactive neurons can trigger a burst; second, the burst occurs after sufficient time for percolation of network interactions to reach threshold. Kam and Feldman (Kam et al., 2013a) divorced preinspiratory activity from bursts, showing that rhythmic burstlets remained in their absence, and argued that burstlets represent the rhythmogenic substrate.

We also observed preBötC field activity like burstlets absent XII output at all [K^+^]_o_ levels. Manipulating network excitability detaches the preinspiratory and inspiratory components of preBötC burst and affects their prevalence. Field and whole-cell recordings from preBötC neurons showed that burstlet rise time, duration, and amplitude match preinspiratory activity. These data affirm the hypothesis that both burstlets and preinspiratory activity share a common rhythmogenic mechanism.

### How many constituent neurons activate during bursts and burstlets?

The frequency of preBötC composite rhythm differed significantly between field recordings and whole-cell recordings at 6 mM [K^+^]_o_. Field recordings reflect activity among many preBötC neurons, whereas whole-cell recordings reflect just one constituent preBötC neuron. Two things change as excitability decreases. First, fewer neurons participate in burstlets but field recordings still detect the collective events. Second, any neuron singled out for whole-cell recording is less likely to be part of the burstlet-active subpopulation. The initial burstlet report showed that ∼89% of preBötC neurons active during bursts also participate in burstlets. Here, using photonics to monitor ∼62 *Dbx1*-derived inspiratory burst-active neurons, we found ∼20% participate in burstlets. We conclude using our whole-cell recordings and photonic recordings that the subset of burstlet-active neurons is inconstant and appears lower than 89%.

The estimated size of the rhythmogenic population is 560–650 preBötC neurons (Hayes et al., 2012; Wang et al., 2014) so the burstlet-active subpopulation numbers between 112–130 (20%) and 500–580 (89%). That seemingly large range can explain why burstlet amplitude varies with [K^+^]_o_: increasing excitability can recruit potentially hundreds of additional constituent preBötC neurons to the burstlet-active subpopulation. Whether or not the fraction of burstlet-active preBötC neurons is closer to 20% or 89%, very few (<10) coactive preBötC neurons can trigger full bursts and motor output (Kam et al., 2013b; Sun et al., 2019) so the relative fraction of burstlet-active neurons may not be a critical parameter governing network activity.

### Burstlet mechanism: network oscillator that depends on recurrent synaptic excitation

Here, f_preBötC_ increased monotonically with [K^+^]_o_. Because [K^+^]_o_ modulates network excitability via direct influence on baseline membrane potential in preBötC constituent neurons, we conclude that preBötC composite rhythm is voltage dependent. In contrast, the initial burstlet report showed no statistically significant disparity between f_preBötC_ at 6 vs 9 mM [K^+^]_o_, yet there was a disparity for 3 mM versus either 6 or 9 mM. This left the question open as to whether burstlet rhythm might be some form of synchronized voltage-independent biochemical oscillator in constituent neurons. Monotonically increasing f_preBötC_ as a function of [K^+^]_o_ rules out that possibility. A significant difference in f_preBötC_ and f_XII_, at 3 and 4 mM [K^+^]_o_, suggests that burstlets maintain the preBötC rhythm. These data imply that burstlets are rhythmogenic in nature, but that concept of rhythmogenicity has not been proven and awaits definitive testing.

So, what mechanism does give rise to burstlets? Our whole-cell recordings show temporal summation of EPSPs during burstlets. We held membrane potential at –60 mV, which imposes steady-state deactivation of the persistent Na^+^ current (Del Negro et al., 2002; Ptak et al., 2005; Yamanishi et al., 2018). Therefore, burstlets do not reflect voltage-dependent bursting properties and do appear to reflect recurrent synaptic excitation.

In general, network oscillators (distinct from pacemaker or inhibition-based models) rely on recurrent excitation among constituent rhythmogenic neurons (Grillner, 2006; Grillner and El Manira, 2020). Modifying the neuronal excitability influences the relative fraction of spontaneously active neurons in the network, and, for silent neurons, the proximity of baseline membrane potential to spike threshold. Increasing excitability therefore magnifies the number of neurons interacting, facilitates synaptic drive summation, and accelerates the process of recurrent excitation to directly influence frequency. We conclude that burstlets are not only rhythmogenic, but also follow dynamics of recurrent excitation, i.e., a network oscillator model of rhythmogenesis.

### Frequency control differs for sighs compared to burstlets and bursts

The preBötC can generate burstlets, inspiratory bursts, and sigh-related bursts (Lieske et al., 2000; Ruangkittisakul et al., 2008). Here, f_sigh_ increased as the [K^+^]_o_ level increased, which at face value suggests voltage dependence (akin to f_preBötC_ and f_XII_ as argued above). However, f_sigh_ is an order of magnitude lower than f_preBötC_ and f_XII_. Further, the [K^+^]_o_-dependent increase in f_sigh_ is 20 times less steep than that of f_preBötC_ or f_XII_. These observations suggest that the mechanism for frequency control of the composite preBötC rhythm and the XII motor output do not similarly apply to sigh rhythm. We propose that sigh frequency control is not voltage dependent like burstlets and inspiratory bursts, which implicates a biochemical oscillator for sigh rhythms that interacts with the network oscillator underlying burstlets and inspiratory bursts to bring about the less-steep f_sigh_ versus [K^+^]_o_ curve. We cannot yet specify how the sigh rhythm is generated; it is beyond the scope of this paper.

### Pattern generation

Burstlet amplitude depends on the excitability too. Above, we inferred that additional constituent neurons (perhaps ∼100 s) are recruited as excitability increases. In contrast, the amplitude of preBötC bursts and XII motor output are not voltage dependent across [K^+^]_o_ levels. Although burstlet amplitude changes with excitability, the rise time, fall time, and duration of burstlet events do not vary with [K^+^]_o_ levels. First, we conclude that the network dynamics that recruit burstlet-active neurons, and govern the temporal evolution of burstlets, are largely the same at different levels of excitability. Second, we conclude that preBötC activity, once passing threshold, triggers a cascade that activates all (or nearly all) preBötC neurons, and also activates premotor and motor neurons. That cascade probably depends on synaptic connectivity among pattern-related preBötC neurons and premotor neurons outside of the preBötC, but not preBötC excitability per se.

We cannot yet specify how activity during the preinspiratory phase reaches a threshold for burst generation. It may have to do with a quorum: a certain number of rhythmogenic interneurons must be active (i.e., spiking) to trigger an irreversible cascade that ostensibly activates all (or nearly all) preBötC neurons. Or, it may have to do with synchrony: a certain number of rhythmogenic interneurons must be spiking in sync to trigger that cascade (Ashhad and Feldman, 2019). The former focuses on mass action of constituent interneurons wherein temporal precision is inconsequential. The latter focuses on phasic precision rather than mass action. Our present data cannot distinguish which mechanism is at work but given that burstlet amplitude is voltage-dependent and the number of constituent neurons participating in burstlets may vary at any given level of excitability, the quorum model seems less feasible.

There is an existing framework for understanding both rhythm and pattern generation of the preBötC. *Dbx1*-derived preBötC neurons are inspiratory rhythmogenic (Bouvier et al., 2010; Gray et al., 2010; Wang et al., 2014; Cui et al., 2016; Koizumi et al., 2016; Vann et al., 2016, 2018; Baertsch et al., 2018), playing key preinspiratory and burst-generating roles (Picardo et al., 2013; Cui et al., 2016), and some serving exclusively premotor function (Revill et al., 2015). It may be possible to identify subsets of predominantly rhythmogenic versus predominantly premotor or pattern-related *Dbx1*-derived preBötC neurons based on intrinsic membrane properties (Picardo et al., 2013) or neuropeptide somatostatin expression (Cui et al., 2016). Those two classification schemes are not mutually exclusive because many somatostatin- and somatostatin receptor-expressing preBötC neurons are *Dbx1*-derived (Gray et al., 2010). It is also possible that particular ion channels serve in a pattern-related capacity within core rhythmogenic neurons. For example, transient receptor potential (Trp) channels in *Dbx1*-derived preBötC neurons whose activation amplifies inspiratory burst magnitude (Koizumi et al., 2018; Picardo et al., 2019) also maintain the tidal volume of inspiratory breaths in unanesthetized adult mice (Picardo et al., 2019).

Our results affirm the ideas presented in the burstlet hypothesis (Kam et al., 2013a) that rhythm and pattern generation are discrete processes, which nevertheless both begin in the preBötC. Burstlets, subthreshold from the standpoint of motor discharge, appear to reflect the core rhythmogenic mechanism involving recurrent synaptic excitation.

## Synthesis

Reviewing Editor: Douglas Bayliss, University of Virginia School of Medicine Decisions are customarily a result of the Reviewing Editor and the peer reviewers coming together and discussing their recommendations until a consensus is reached. When revisions are invited, a fact-based synthesis statement explaining their decision and outlining what is needed to prepare a revision will be listed below. The following reviewer(s) agreed to reveal their identity: Arlette Kolta, Fernando Peña-Ortega. Note: If this manuscript was transferred from JNeurosci and a decision was made to accept the manuscript without peer review, a brief statement to this effect will instead be what is listed below.

This manuscript was seen by two reviewers, who determined that it represents a valuable confirmation of previous work, even if relatively narrow in scope and providing only a modest conceptual advance. They also shared concerns with several aspects of the experimental design, data analysis and presentation, and with some of the associated interpretations. The full critiques are provided further below.

Here, we draw your attention to some key issues. In this replication study, it will be important to include some extracellular recordings similar to those done in the original work, in order to try to account for some discrepancies in the incidence of cells recruited during 'burstlets'. In addition, simultaneous recordings of cellular and population level activity, with additional quantitative analyses, are requested to properly validate and characterize identified 'burstlets' and their presumed relationship to rhythm- or pattern-generating circuit activity. A further shared interpretive concern involved advising some caution in conflating changes in [K]e and network excitability with voltage dependence.

The original Reviewer comments are appended below.

Reviewer #1

Advances the Field

The paper mostly reproduces findings from another paper and adds very little new findings. However, it does confirm previous findings from another (presumably) group. I'm not sure if this, by itself, suffice to guarantee publication. There are no major flaws in the experiments per se, but interpretation of the results should be revised.

Comments to the Authors

In this paper, the authors use imaging and electrophysiological recordings (whole-cell, and field recordings) in reduced preparations of the preBötzinger complex (preBötC) and the hypoglossal circuits, to test the burstlet hypothesis proposed by Feldman's group in 2013. This hypothesis posits that the mechanisms underlying the inspiratory rhythm and the pattern generation are distinct but co-exist within the preBötC. The authors were able to reproduce the findings of Kam and Feldman. Besides this additional support to the “burstlet” hypothesis, this study adds little but a few more findings to those previously reported. These include the fact that the preBötC composite rhythm increases linearly with the extracellular K+ concentration ([K+]o ; representing increased excitability) and that intracellular recordings show that “burslets” reflect temporal summation of EPSPs.

The paper is generally well written, but the amount of work and data in it do not represent significant advancement in the field. There are also many assumptions that need to be better explained or motivated. For instances, the effects of increasing [K+]o are often equated to voltage-dependency without any proof. Increasing [K+]o may have an effect on many other components in a circuitry, whether it is other neurons, glia, conductances...etc. Therefore, I strongly recommend changing voltage-dependency everywhere to either increased excitability or simply increased [K+]o. With regard to [K+]o, a concentration of 9 mM is used to approximate a state of higher excitability, but is this physiological or shown to exist under physiological conditions? Another important issue with this paper as it is presented is the absence of raw records to support some assertions. For instances, in intracellular records, some events (summated EPSPs) are described as reflecting burstlets, but simultaneous field recordings in preBötC are not shown (Fig 5). Therefore, we do not know if they truly represent burstlets. Similarly, in Fig 6-8 it would have been interesting to see the raw traces to see if there is a correlation between the number of cells and the amplitude of the burstlets or timing between the events. Finally, another disturbing assumption, that is often repeated throughout the paper is that burstlets represent the rhythmogenic component of the circuitry, whereas bursts represent the patterning component, but there is no rhythm analysis on the burstlets with any of the conventional methods (power spectrum analysis...etc). Kam and Feldman had at least done some analysis of the rhythm. Thus, overall this paper adds little to the previous findings.

Minor:

Vertical calibration is lacking in Fig 1A and 4A and B. Some events presented as burstlets are not very convincing given the high variability of the noise in the baseline records.

Horizontal calibration is lacking in Fig 5A

Legend of Fig 8 should provide more details. It does not even state what is presented. Also, what are the wavelets of activity in Neurone 13 under 9mM?

Reviewer #2

Advances the Field

This study confirms the report by Kam et al., 2013.

Comments to the Authors

The study “Evaluating the burstlet theory of inspiratory rhythm and pattern generation” is a thorough and complementary study, which mostly confirms and expands the study by Kam et al., 2013. Regarding the replication of the previous study, the authors made a great effort on replicating most of the findings on this very important. However, I there are two general points that I would like to comment on the additional experiments:

--This study has used whole-cell patch clamp recordings to evaluate the presence of burstlets at the single-cell level, which has rendered differences in the proportion of cells recruited during them (20% in this study vs 89% found by Kam et al., 2013). Considering that the excitability of the recorded cell can be changed by the whole-cell configuration (mainly by changes in the intracellular milieu), I suggest that a similar experimental approach as the one used by Kam et al should be used too (extracellular recordings).

--Related to the previous comments, in this study “burstlets” were detected at the single cell level (burst of synaptic potentials), but those burstlets were not correlated to actual burstlets supposedly occurring simultaneously at the preBötC population level. I think this should be addressed systematically. First of all, simultaneous recordings from preBötC neurons and preBötC population activity are required to stablish the correlation of single-cell busrtlets and population burstlets. In addition, quantification of spontaneous synaptic activity received by individual preBötC neurons should be performed to determine, whether or not, the single-cell burstlets are events that belong to the population of spontaneous synaptic potentials or burstlets are synaptic events that have a significantly bigger amplitude, frequency and/or duration that the background spontaneous synaptic potentials.

Next, I will enumerate some other suggestions:

-I like to comment that the description of the burstlet detection does not necessarily match with the burstlets shown in the figures. There are clearly a couple of bursts in the upper traces from Figure 4A that, by comparison with the others, should meet the criteria but were not included. I specifically refer to the likely burstlet in the 4A upper trace (perhaps two) located between the first one identified with an x and the one identified by the gray rectangle. Histograms with the distribution of some pattern characteristics of the detected burstlets should be provided (amplitude, duration, rise- and decay-time) to better clarify burstlet selection and pattern.

-In figure 4A it can also be seen that burstlets could be detected in the hypoglossal recordings. In fact, some XII burstlets seem to be synchronous with preBötC burstlets. So, I am wondering if preBötC burstlets can be reflected in XII burstlets (also seen in Fig. 1A, for the only burstlet detected at 9 mM)? Or, is it possible that XII could show independent burstlets (as in Fig. 1A at 5 mM)?

- A proper quantitative assessment of burstlet coupling (i.e cross-correlation or burstlet-triggered average) between both preBötCs should be included.

-A detailed description of how bursts and burstlets were detected and their characteristics quantified is needed. For instance, please explain how burst onset and end were detected. How burst area was quantified? If fact, providing histograms of these quantification could help to get a sense of the variety of burstlets' and burst's shapes.

-It was not clear to me how RMS smoothing was performed. Was it done through a differential amplifier? How so? Please, explain in detail.

-Related with the previous comment; once providing a description of how the RMS smoothed signal was obtained, it would be good to explain how those recordings were calibrated (i.e. amplitude is shown in mV units in Table 1). Then, if calibration is possible, please include amplitude calibration bars in all figures.

-Considering that this is a confirmatory study, I did not find any mention/characterization of “doublets” described by Kam et al., 2013 (also detected in some of the recordings in this study). It would be good to deal with this issue in the Introduction and/or Discussion.

-There is a frequent affirmation in the manuscript that burstlets and the composite rhythm are “voltage dependent”. However, the relationship between voltage and some of the parameters is not thoroughly described. I get the impression that most of the relationships are not linear (Fig. 1B, 2A, 2B). In fact, there seems to be all-or-nothing relationships (Fig. 1B, 2A) or there seems to be a threshold for some changes (Fig. 1B, 2A, 2B). I think, this should be discussed and the voltage-dependency of some of the described phenomena be more precisely described and discussed.

-The variability of the bursts is quantified regarding frequency but not amplitude. I consider that quantifying this last parameter can also offer relevant information.

-Considering that the amplitude of burstlets change when K+ concentration is modified and that this has been associated to voltage dependent changes in excitability, I am wondering if the background network activity also changes (which should be evaluated) and, therefore, the threshold for burstlet detection could change as well (which should be acknowledged, if that is the case).

-Is the rising phase of burstlets changing upon modifications in K+ concentration?

-It would be helpful to include labels for the statistical difference(s) in the graphs of Figures 1B; 2; 3; 4C and 7 (as actually included in Fig. 6).

-A clear definition of a “burstlet-active” neuron is required. I assume that “burstlet-active” neurons are the ones exhibiting single-cell burstlets. However, as already mentioned, it would be good to actually record preBötC population burstlets and used them to perform population burstlet-triggered average in single preBötC cells to evaluate if, in fact, the number of cells receiving inputs during population burstlets is actually low, or not.

-A definition of “burst-active” neurons is also required.

-Calcium-dependent fluorescence imaging used in this study needs to be “calibrated”. It should be defined if this signal is action potential-dependent or not. If not, it should be defined what kind of subthreshold calcium-dependent fluorescence can be recorded and what characteristics of the signal are related to action potentials generation.

-I am not sure that holding the cells at -60 mV is the best strategy to reveal those neurons that might get activated during burstlets. For neurons exhibiting a more depolarized potential, the likelihood of generation action potentials during population burstlets was artificially reduced. Thus, I suggest recording the population preBötC activity simultaneously with single-cells left at their original membrane potential, which could reveal more “burstlet-active” neurons, or not.

-Please, include the quantification of XII activity in Figure 2.

-The formats in the references list should be revised. At least, I found two different abbreviations for the J Neurosci.

-Please acknowledge that the R2 of the linear regression relating K+ concentration and burstlet amplitude is too low and provide an interpretation of it.

-For the quantifications provided in Figure 7 I have some suggestions:

a) Provide the standard deviation or the standard error of each measurement.

b) Indicate those experiments in which the No. of active neurons were determined in the same slice (if that is the case).

c) Indicate, if that is the case, whether the No. of active neurons, mainly during bursts, is the maximum number of respiratory-related cells, or it is the average. Are there some bursts exhibiting more active neurons than the average? If so, it would be best to show the data of Fig. 7 as % of maximal number of respiratory cells.

-Finally, in Figure 8B, cell #14 has a change in fluorescence beyond background which was not selected as a burstlet; can you provide the reason why this fluorescence change was excluded?

## Author Response

Synthesis of Reviews:

Computational Neuroscience Model Code Accessibility Comments for Author (Required):

N/A

Significance Statement Comments for Author (Required):

N/A

Comments on the Visual Abstract for Author (Required):

N/A

Synthesis Statement for Author (Required):

This manuscript was seen by two reviewers, who determined that it represents a valuable confirmation of previous work, even if relatively narrow in scope and providing only a modest conceptual advance. They also shared concerns with several aspects of the experimental design, data analysis and presentation, and with some of the associated interpretations. The full critiques are provided further below.

We thank the referees and the Reviewing Editor for a supportive assessment of the work overall. Yes, we agree, the work is confirmation of previous work (which is valuable given the widely acknowledged reliability and replicability crisis in neuroscience). Yes, we agree that the scope is narrow. However, we respectfully push back on the “modest conceptual advance”. Respiratory neurobiologists have sought to understand rhythm generation in the preBötzinger complex (preBötC) since 1991 and three major canonical models (pacemakers, synaptic inhibition, and the group pacemaker) have been found lacking. Kam & Feldman's idea that inspiratory rhythm depends on 'burstlets' - subthreshold from the standpoint of motor output - is a radical idea and so far, no one has attempted to evaluate it! We argue that the conceptual importance of testing and evaluating burstlet theory has enormous implications for our field and for central pattern generators (CPGs).

Here, we draw your attention to some key issues. In this replication study, it will be important to include some extracellular recordings similar to those done in the original work, in order to try to account for some discrepancies in the incidence of cells recruited during 'burstlets'. In addition, simultaneous recordings of cellular and population level activity, with additional quantitative analyses, are requested to properly validate and characterize identified 'burstlets' and their presumed relationship to rhythm- or pattern-generating circuit activity. A further shared interpretive concern involved advising some caution in conflating changes in [K]e and network excitability with voltage dependence.

We reassess the incidence of neurons active during burstlets (see new Fig. 6) using triple recordings. We performed additional quantitative analyses, which appear throughout the manuscript. Also, we modified our narrative such that changes in [K+]o are mapped to general “excitability” rather than strictly voltage dependence.

The original Reviewer comments are appended below.

Reviewer #1

Advances the Field

The paper mostly reproduces findings from another paper and adds very little new findings. However, it does confirm previous findings from another (presumably) group. I'm not sure if this, by itself, suffice to guarantee publication. There are no major flaws in the experiments per se, but interpretation of the results should be revised.

We thank Reviewer #1 for a supportive assessment of the work overall.

Comments to the Authors

In this paper, the authors use imaging and electrophysiological recordings (whole-cell, and field recordings) in reduced preparations of the preBötzinger complex (preBötC) and the hypoglossal circuits, to test the burstlet hypothesis proposed by Feldman's group in 2013. This hypothesis posits that the mechanisms underlying the inspiratory rhythm and the pattern generation are distinct but co-exist within the preBötC. The authors were able to reproduce the findings of Kam and Feldman. Besides this additional support to the “burstlet” hypothesis, this study adds little but a few more findings to those previously reported. These include the fact that the preBötC composite rhythm increases linearly with the extracellular K+ concentration ([K+]o ; representing increased excitability) and that intracellular recordings show that “burslets” reflect temporal summation of EPSPs.

The paper is generally well written, but the amount of work and data in it do not represent significant advancement in the field. There are also many assumptions that need to be better explained or motivated. For instances, the effects of increasing [K+]o are often equated to voltage-dependency without any proof. Increasing [K+]o may have an effect on many other components in a circuitry, whether it is other neurons, glia, conductances...etc. Therefore, I strongly recommend changing voltage-dependency everywhere to either increased excitability or simply increased [K+]o. [break by authors]

We changed voltage dependence to excitability throughout the manuscript (too many instances to list here). Nevertheless, because manipulating [K+]o directly impacts baseline membrane potential as described by the Goldman-Hodgkin-Katz (GHK) equation, changes to [K+]o do imply a certain degree of voltage dependence. We interpret our data in terms of voltage dependence in the Discussion (Page 17 Line 360-362).

...With regard to [K+]o, a concentration of 9 mM is used to approximate a state of higher excitability, but is this physiological or shown to exist under physiological conditions? [break by authors]

9 mM [K+]o is not physiological, but elevated [K+]o is a well-established tool to augment excitability in vitro. Isolating the preBötC in slice preparations necessarily removes all the tonic excitatory drive from rostral brainstem, e.g., the chemosensitive retrotrapezoid nucleus (RTN), the pons (A2/C2, A5, parabrachial nucleus), and the midbrain (see: Yang & Feldman, J Comp Neurol, 526: 1389, 2018; Smith et al. J Comp Neurol 281: 69, 1989). Thus, isolated in slices, the preBötC remains quiescent (arrhythmic). It is now widely accepted that slice studies elevate the [K+]o to 8-9 mM to compensate for loss of tonic drive (originally shown in Smith et al., Science, 254: 726, 1991; and most recently reviewed by Funk & Greer, Respir Physiol Neurobiol, 186:236, 2013), which enables stable rhythmic activity lasting several hours.

Note that rhythmic frequency in slices reaches a plateau for [K+]o above 9 mM (Del Negro et al., J Physiol, 587: 1217, 2009; Johnson et al., J Neurophysiol, 85: 1772, 2001). The other option is to reduce [Ca2+]o to 0.8-1.0 mM, which has the same impact of elevating excitability (Ruangkittisakul et al., Respir Physiol Neurobiol, 175:37, 2011).

The respiratory neurobiology community adopted elevated [K+]o to boost excitability for its in vitro preparations whereas the locomotor community, studying lumbar spinal cord segments ~L2-L5, adopted a cocktail of NMDA + 5HT, which substitutes for the lack of descending drive. In both cases one can easily argue that the manipulations are non-physiological but justifiable to evoke central pattern generator functionality.

...Another important issue with this paper as it is presented is the absence of raw records to support some assertions. For instances, in intracellular records, some events (summated EPSPs) are described as reflecting burstlets, but simultaneous field recordings in preBötC are not shown (Fig 5). Therefore, we do not know if they truly represent burstlets. [break by authors]

We added new experiments where we perform simultaneous whole-cell current-clamp recording, preBötC field recording, and XII nerve recording to demonstrate that the summated EPSPs reflect population burstlets (Fig. 6C).

...Similarly, in Fig 6-8 it would have been interesting to see the raw traces to see if there is a correlation between the number of cells and the amplitude of the burstlets or timing between the events. [break by authors]

Good idea but respectfully it is not feasible to perform extracellular field recordings and 2-photon imaging simultaneously. The geometry and working distance of the objective are prohibitive, but furthermore, the presence of a recording pipette obscures the optics and profoundly reduces the number of neurons visible in the imaging plane.

...Finally, another disturbing assumption, that is often repeated throughout the paper is that burstlets represent the rhythmogenic component of the circuitry, whereas bursts represent the patterning component, but there is no rhythm analysis on the burstlets with any of the conventional methods (power spectrum analysis...etc). Kam and Feldman had at least done some analysis of the rhythm. [break by authors]

Kam & Feldman suggest the rhythmic nature of burstlets first because the preBötC rhythm persists at 6 mM and 3 mM [K+]o, whereas the XII motor output rhythm slows down significantly (and sometimes stops at 3 mM). We added paired-t test results at 3 and 4 mM [K+]o comparing preBötC rhythm and XII motor output rhythm and we arrive at the same conclusion as Kam & Feldman that burstlets sustain preBötC rhythm even when the XII motor output slows down significantly or stops (updated Fig. 2A, Page 10 Line 207-211).

Kam & Feldman do many subsequent tests that suggest the rhythmogenic nature of burstlets, most of which we replicated. None of the tests done by Kam & Feldman prove that burstlets are rhythmogenic, but it is an argument that they make, and we find corroborative support for, based on “analysis of the rhythm” via multiple experiments.

Respectfully, there are no power spectra in Kam et al., J Neurosci 33: 9235, 2013. We remain unconvinced that power spectra or wavelet analysis would have added anything to their study or ours because those tools dissect the frequency components of the signals, but their interpretation is subjective, not dispositive regarding the source of different frequency components.

The only proof that burstlets are rhythmogenic will come about when we find how to selectively perturb them by manipulating the subpopulation of burstlet-active neurons (perhaps on the basis of peptide or transcription factor expression), but that test is beyond the scope of this paper.

...Thus, overall this paper adds little to the previous findings.

We respectfully push back. Our paper shows, for the first time, how burstlets evolve intracellularly in Dbx1-derived preBötC neurons, which are rhythmogenic. For all the merits of Kam & Feldman's 2013 report, there were no intracellular data therein. Our whole-cell current-clamp recordings demonstrate: (1) the similarity between burstlets and preinspiratory activity at an intracellular level, thus underpinning an important claim in the burstlet theory that burstlet and preinspiratory activity are analogous. (2) We further show that burstlets reflect summation of EPSPs rather than, for example, the recruitment of voltage-dependent membrane currents (like persistent Na+ current). (3) By adding experiments at each level between 3-9 mM [K+]o we tested the relation of rhythm- and pattern- generating mechanisms with respect to the excitability in the preBötC network for more thoroughly than Kam & Feldman (2013) did. We are not disparaging them, merely pointing out how we build upon their report. Additionally, (4) using photonic recordings we were able to show that an inconstant subset of Dbx1-derived preBötC neurons participates during burstlets and this subset is 20-30% of the Dbx1-derived preBötC inspiratory neurons. There were no multi-photon imaging experiments in Kam & Feldman's seminal (2013) study. Therefore, we argue that we have added a lot!

Minor:

Vertical calibration is lacking in Fig 1A and 4A and B.

Those figures show preBötC field and XII nerve recordings. The amplitude has no biophysical meaning; it varies based on the gain of the differential amplifier, the seal of the electrode on nerve or field tissue, and the diameter of the electrode. Unlike whole-cell current-clamp recording, vertical calibrations in those figures do not provide any interpretable information. We would also like to point out none of the figures with field or XII nerve recording in Kam & Feldman's paper have vertical calibration bars.

Some events presented as burstlets are not very convincing given the high variability of the noise in the baseline records.

We agree that the burstlet events can be confused with noise. Thus, we established that the only events identified as burstlets crossed our threshold detection criterion, which ensures that their peaks exceed the 95% confidence intervals (CIs) of baseline noise and thus are unlikely to be spurious noise-related events.

Horizontal calibration is lacking in Fig 5A.

We added the horizontal (time) calibration bar in Fig. 5A.

Legend of Fig 8 should provide more details. It does not even state what is presented.

We added more detail to the legend for Fig. 8, indicating that it reflects 2-photon imaging in the population of Dbx1-derived preBötC neurons.

Also, what are the wavelets of activity in Neurone 13 under 9mM?

We consider the activity as noise, not respiratory-related.

Reviewer #2

Advances the Field

This study confirms the report by Kam et al., 2013.

Comments to the Authors

The study “Evaluating the burstlet theory of inspiratory rhythm and pattern generation” is a thorough and complementary study, which mostly confirms and expands the study by Kam et al., 2013. Regarding the replication of the previous study, the authors made a great effort on replicating most of the findings on this very important. However, I there are two general points that I would like to comment on the additional experiments:

We thank Reviewer #2 for a supportive assessment of the work overall.

-- This study has used whole-cell patch clamp recordings to evaluate the presence of burstlets at the single-cell level, which has rendered differences in the proportion of cells recruited during them (20% in this study vs 89% found by Kam et al., 2013). Considering that the excitability of the recorded cell can be changed by the whole-cell configuration (mainly by changes in the intracellular milieu), I suggest that a similar experimental approach as the one used by Kam et al should be used too (extracellular recordings).

Whole-cell and extracellular recording methods have advantages and disadvantages. While it is true that the whole-cell configuration dialyzes the intracellular milieu, extracellular recordings only report the occurrence of action potentials and the waveform itself does not carry any other information. Therefore, subthreshold potentials due to the summation of EPSPs that influence the evolution of burstlets, but without producing an action potential, remain hidden.

Therefore, we respectfully argue that intracellular recording is always superior because it captures subthreshold potentials underlying burstlets. The disadvantages of whole-cell dialysis are offset by supplying EGTA to buffer Ca2+, HEPES to buffer the pH, as well as ATP and GTP for second messenger systems via the patch pipette solution. Also, we would like to point out that we use 2-photon recordings to reach the conclusion that 20%, and not 89%, of inspiratory neurons are recruited during burstlet and those photonically recorded neurons are not perturbed: neither dialyzed by patch pipette solution nor membrane-permeable Ca2+ dyes.

-- Related to the previous comments, in this study “burstlets” were detected at the single cell level (burst of synaptic potentials), but those burstlets were not correlated to actual burstlets supposedly occurring simultaneously at the preBötC population level. I think this should be addressed systematically. First of all, simultaneous recordings from preBötC neurons and preBötC population activity are required to establish the correlation of single-cell burstlets and population burstlets. [break by authors]

We performed simultaneous triple recordings from Dbx1-derived preBötC neurons and preBötC population activity at 6 mM [K+]o, with XII motor output, to now show that the burstlets identified in the intracellular recording correspond to population burstlets (see revised Fig. 6A,C).

...In addition, quantification of spontaneous synaptic activity received by individual preBötC neurons should be performed to determine, whether or not, the single-cell burstlets are events that belong to the population of spontaneous synaptic potentials or burstlets are synaptic events that have a significantly bigger amplitude, frequency and/or duration that the background spontaneous synaptic potentials.

Of the two possibilities summarized by the reviewer, burstlets are the latter: synaptic events of significantly larger amplitude compared to background spontaneous synaptic potentials. We quantified the mean EPSP amplitude and duration for spontaneous EPSPs that occur during interburst (or inter-burstlet) interval and compared them to the mean amplitude and duration of burstlets. The unpaired t-test was significant in both cases (p = 0.022 and p = 0.004, respectively). Because the EPSPs temporally summate during burstlets we could not analyze the amplitude or duration of any individual EPSP in isolation during burstlets. So, the statistically significant findings above simply show that burstlets are different than spontaneous EPSPs during the interburst (or inter-burstlet) interval. That analysis is consistent with present analytic framework of our manuscript whereby we call out burstlets as coherent events, distinct from baseline noise (or spontaneous EPSPs).

Additionally, our triple recordings also show that the EPSPs identified as single-cell burstlets during intracellular recording coincide with population burstlets. These data imply that the single-cell burstlets are collective synaptic events and so it is not surprising that they exhibit significantly larger amplitude and duration than spontaneous EPSPs that occur between burstlet or burst cycles.

Next, I will enumerate some other suggestions:

-I like to comment that the description of the burstlet detection does not necessarily match with the burstlets shown in the figures. There are clearly a couple of bursts in the upper traces from Figure 4A that, by comparison with the others, should meet the criteria but were not included. I specifically refer to the likely burstlet in the 4A upper trace (perhaps two) located between the first one identified with an x and the one identified by the gray rectangle. [break by authors]

We appreciate Reviewer #2's careful examination of our data but the activity in the upper trace of Fig. 4A does not cross the threshold for burstlet detection, i.e., 95% confidence intervals (CIs) for the distribution of baseline fluctuations (noise) for the preBötC population recording. Thus, although there appear to be burstlet peaks, we cannot consider them so, and thus they are not marked as burstlets nor analyzed as such.

...Histograms with the distribution of some pattern characteristics of the detected burstlets should be provided (amplitude, duration, rise- and decay-time) to better clarify burstlet selection and pattern.

We report the amplitude of burstlets (Fig. 3B) and the change in amplitude as the excitability in the preBötC network changes (Fig. 3B and Table 1). We added a table with pattern characteristics suggest by the reviewer, which reports duration, rise-time and decay-time of burstlets at each excitability level (new Table 2).

- In figure 4A it can also be seen that burstlets could be detected in the hypoglossal recordings. In fact, some XII burstlets seem to be synchronous with preBötC burstlets. So, I am wondering if preBötC burstlets can be reflected in XII burstlets (also seen in Fig. 1A, for the only burstlet detected at 9 mM)? Or, is it possible that XII could show independent burstlets (as in Fig. 1A at 5 mM)?

Kam & Feldman (2013) pointed out that manipulations of excitability can reveal XII burstlets in vitro (their Fig. 4C,D), which may also occur in vivo after vagotomy, disinhibition, and application of bombesin (their Fig. 7A). Therefore, we are open to the idea of some activity in XII output that is synchronous with preBötC burstlets. However, in this case the activity did not cross the threshold detection of the 95% CIs for the distribution of baseline fluctuations (noise) for the XII nerve recording. Thus, these activities cannot be considered XII burstlets by conventional statistical criteria.

- A proper quantitative assessment of burstlet coupling (i.e cross-correlation or burstlet-triggered average) between both preBötCs should be included.

We performed cross-correlation of burstlets for bilateral preBötC recordings. The Pearson's r correlation mean value for 3 mM and 6 mM [K+]o are 0.734 {plus minus} 0.127 and 0.747 {plus minus} 0.079 respectively. The Pearson's r varies from -1 to +1, where +1 implies highly correlated. Thus, bilateral burstlets are more than 70% correlated. Below is an example of the cross correlogram at 3 mM [K+]o.

We use bilateral recordings to see if the burstlets are bilaterally synchronous or not, and as stated in the paper 92% {plus minus} 7% and 97% {plus minus} 4% (Page 11 Line 239-240) of burstlets, at 6 and 3 mM [K+]o respectively, are synchronous. Because the correlation statistics results do not provide any new information not already contained in the quantification (Page 11 Line 239-240) we prefer not to add the additional statistics to our narrative.
